# *De novo* Cloning and Annotation of Genes Associated with Immunity, Detoxification and Energy Metabolism from the Fat Body of the Oriental Fruit Fly, *Bactrocera dorsalis*

**DOI:** 10.1371/journal.pone.0094470

**Published:** 2014-04-07

**Authors:** Wen-Jia Yang, Guo-Rui Yuan, Lin Cong, Yi-Fei Xie, Jin-Jun Wang

**Affiliations:** Key Laboratory of Entomology and Pest Control Engineering, College of Plant Protection, Southwest University, Chongqing, China; Zhejiang University, China

## Abstract

The oriental fruit fly, *Bactrocera dorsalis*, is a destructive pest in tropical and subtropical areas. In this study, we performed transcriptome-wide analysis of the fat body of *B. dorsalis* and obtained more than 59 million sequencing reads, which were assembled into 27,787 unigenes with an average length of 591 bp. Among them, 17,442 (62.8%) unigenes matched known proteins in the NCBI database. The assembled sequences were further annotated with gene ontology, cluster of orthologous group terms, and Kyoto encyclopedia of genes and genomes. In depth analysis was performed to identify genes putatively involved in immunity, detoxification, and energy metabolism. Many new genes were identified including serpins, peptidoglycan recognition proteins and defensins, which were potentially linked to immune defense. Many detoxification genes were identified, including cytochrome P450s, glutathione S-transferases and ATP-binding cassette (ABC) transporters. Many new transcripts possibly involved in energy metabolism, including fatty acid desaturases, lipases, alpha amylases, and trehalose-6-phosphate synthases, were identified. Moreover, we randomly selected some genes to examine their expression patterns in different tissues by quantitative real-time PCR, which indicated that some genes exhibited fat body-specific expression in *B. dorsalis*. The identification of a numerous transcripts in the fat body of *B. dorsalis* laid the foundation for future studies on the functions of these genes.

## Introduction

The oriental fruit fly, *Bactrocera dorsalis* (Hendel) (Diptera: Tephritidae), is a highly destructive pest, and is distributed widely in tropical and subtropical areas around the world. It can feed and reproduce on more than 250 species of plants, causing enormous losses in many economically important agriculture products, such as fruits and vegetables [1]. Currently, insecticides are the most effective way to control this pest. However, resistance monitoring programs found that various populations of *B. dorsalis* have become resistant to different kinds of insecticides, resulting in their resurgence and environmental risks [2]. Therefore, more potent and powerful strategies are required to manage this pest. In recent years, RNA interference (RNAi)-based technology has shown great potential in pest control by silencing vital genes [3], [4]. For this method, abundant genomic resources are essential and necessary to identify suitable target genes for RNAi [5]. Unfortunately, molecular genetic information for *B. dorsalis* is relatively limited, and the entire genome is not yet available, although the analysis is in progress (Bactrobase: http://www.bactrobase.org/). However, transcriptome sequencing of *B. dorsalis* has been performed successfully, and has proved to be an effective method to gather genetic information from either the whole body or the midgut [6], [7]. These data have provided comprehensive gene expression information regarding detoxification, digestion, development and the RNAi mechanism. However, further tissue-specific transcriptome analyses are required to obtain a comprehensive view of the temporal and spatial changes in gene expression related to unique biological features.

The insect fat body is a dynamic tissue whose function is equivalent to the vertebrate liver. It is a major organ involved in various physiological and biological processes, including detoxification, developmental regulation and immunity [8], [9], [10]. Previously, because of technical limitations, molecular studies on the fat body initially focused on characterization of individual genes, particularly those involved in detoxification [11] or energy storage and release [10]. With the development of next generation sequencing (high-throughput deep sequencing) technology, characterization of the entire set of genes expressed in the insect fat body has become feasible. Such works have been conducted in the fruit fly (*Drosophila melanogaster*) [12], the tsetse fly (*Glossina morsitans morsitans*) [8], and the yellow fever mosquito (*Aedes aegypti*) [13], [14]. Despite the importance of this tissue, little is known about the molecular basis of physiological and immunological mechanisms in the fat body of *B. dorsalis*. In this study, we performed high-throughput Illumina Solexa sequencing to acquire a comprehensive view of the genes expressed in the fat body of *B. dorsalis*. We focused particularly on genes related to immune defense, detoxification and energy metabolism because of their importance for insect development. Furthermore, we investigated the gene expression patterns of several key genes in different tissues by quantitative real-time PCR. The results obtained from this study will provide an invaluable resource for future functional studies on fat bodies in *B. dorsalis*.

## Results and Discussion

### Illumina sequencing and reads assembly

Illumina sequencing was used to sequence a cDNA library from the fat body of *B. dorsalis*, and generated 59,158,922 reads with a length of 4,959,025,200 bp (Runs accession number: SRR1026844). To facilitate sequence assembly, the Trinity program assembled these raw reads into 46,444 contigs with an average length of 360 bp. Paired end-joining and gap-filling further assembled the contigs into 27,787 unigenes with an average length of 591 bp (Table 1). The analysis of the length distribution of the unigenes revealed that 4,336 unigenes (15.6%) were more than 1,000 bp, 5,039 unigenes (18.1%) were between 500 and 1,000 bp, and 18,412 unigenes (66.3%) ranged from 100 to 500 bp (Figure S1).

**Table 1:** Sequencing summary for the fat body transcriptome of *Bactrocera dorsalis*.

Sequencing Summary	Fat body specific transcriptome
Total number of reads	59,158,922
Total base pairs (bp)	4,959,025,200
Average read length (bp)	84
Total number of contigs	46,444
Mean length of contigs (bp)	360
N50^d^ of contigs	628
Total number of unigenes	27,787
Mean length of unigenes (bp)	591
N50^d^ of unigenes	945
Sequences with *E*-value <10^−5^	17,442 (62.8%)

For BLASTX annotation, the unigenes were searched against the non-redundant (nr) NCBI nucleotide database. A total of 17,442 unigenes (62.8%) returned positive BLAST results at a cut-off *E*-value of 10^−5^. However, the remaining 10,345 unigenes (37.2%) could not be matched to known genes in the NCBI database, either because of a lack of *B. dorsalis* genome information or their short nucleotide length.

### Sequence homology distribution

The *E*-value distribution of the 17,442 annotated unigenes showed that 40.2% of the sequences had significant homology (*E*-value less than 1.0E^−45^) matching in the NCBI database, while 59.8% of the sequences had *E*-values that ranged from 1.0E^−5^ to 1.0E^−45^ (Figure S2A). The similarity distribution showed that 27.1% of the sequences had significant similarity higher than 80%, followed by 60.4% of the sequences with homology between 40 and 80%; only 12.5% of the sequences had similarity lower than 40% (Figure S2B). For species distribution, the majority of unigenes (72.0%) showed significant similarity with *Drosophila*. Of these, 13.0% of the unigene sequences had top matches (lowest *E*-value) with sequences from *D. virilis*, followed by *D. willistoni* (12.6%), *D. melanogaster* (11.6%), *D. mojavensis* (11.0%), and other *Drosophila* species (Figure S2C). This was in accord with our previously study of transcriptome profiling of the whole body and midgut of *B. dorsalis*, in which over 79% genes were most closely related to *Drosophila* [6], [7]. There were 249 unigene sequences (1.4%) that matched genes from *B. dorsalis* and the majority of these hits matched to cytochrome P450s and glutathione S-transferases (data not shown). In addition, 279 unigenes had a best hit to other insects from the *Bactrocera*, such as *B. oleae* (165 unigenes), *B. cucurbitae* (36), *B. tryoni* (32), *B. papaya* (19), *B. correcta* (18), and *B. minax* (9).

### Functional classification and pathway analysis

**Figure 1:**
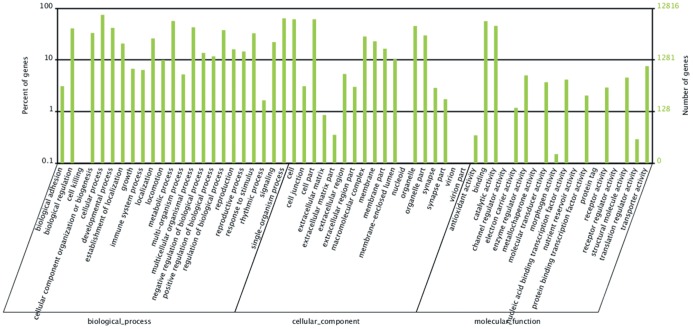
Gene Ontology (GO) classification of unigenes in the fat body of *Bactrocera dorsalis*. 11,575 sequences were annotated within the GO database and summarized in three main categories: biological process, cellular component and molecular function. The left and right y-axes indicate the percentage and number of genes in each category, respectively.

Gene ontology (GO) assignments were used to classify the functions of the predicted unigenes from fat body of *B. dorsalis*. According to the sequence similarity, 12,816 unigenes (46.0% of total) were categorized into 50 functional groups among the three main ontologies (Figure 1). In each of the three main categories (biological process, cellular component, and molecular function) of the GO classification, “Cellular process and Single-organism process”, “Cell or Cell part”, and “Binding and Catalytic activity” terms were the most abundant. In contrast, the “Cell killing” term under the Biological process category was the least abundant, and contained only three unigenes. The unigenes associated with the “virion” and “virion part” under the Cellular component had no more than three unigenes associated with each term. There were also a low percentage of genes from the categories of “metallochaperone activity”, “protein tag”, and “receptor regulator activity”.

**Figure 2:**
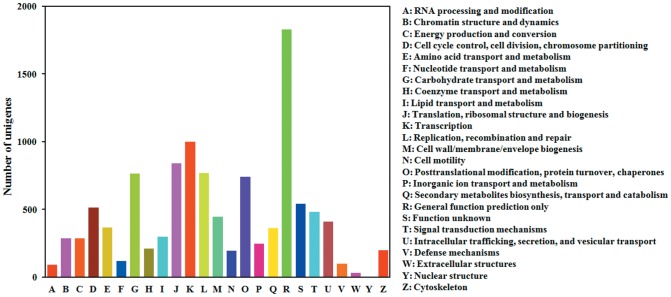
Clusters of Orthologous Groups (COG) classification of unigenes in the fat body of *Bactrocera dorsalis*. Among 17,442 nr hits, 5,185 sequences had a COG classification among the 25 categories.

To further analyze the putative protein functions, the assembled unigenes were compared against Clusters of Orthologous Groups (COG). Out of 17,442 nr hits, 11,122 sequences were assigned a COG classification (Figure 2). Among the 25 COG categories, the cluster for “General function prediction” represented the largest group (1,828, 16.4%), followed by “Transcription” (998, 9.0%), “Translation, ribosomal structure and biogenesis” (839, 7.5%) and “Replication, recombination and repair” (768, 6.9%). In contrast, “Extracellular structures” and “Nuclear structure” represented relatively smaller clusters, containing only 30 and four unigenes, respectively.

Subsequently, Kyoto Encyclopedia of Genes and Genomes (KEGG) pathway analysis was performed on unigenes to identify the biological pathways that are active in the fat body of *B. dorsalis*. There were 11,857 unigenes assigned to 254 KEGG pathways (Table S1). The pathways with most representation among the unique sequences were “Metabolic pathways” (1611 members), “Pathways in cancer” (423 members), and “RNA transport” (400 members). These annotations are helpful to further investigate specific biological processes, functions and pathways occurring in the fat body of *B. dorsalis*.

### Putative immunity-related genes

The insect fat body serves as a barrier between the internal and external environment, and protects the host against infection by microbes and foreign objects [15]. Recent studies found that a significant number of immunity-related genes were highly expressed in this tissue, and participated in triggering an immune response or preventing infection by microbial and protozoan pathogens [8], [16]. These genes are considered as the potential targets for pest control, and their specific sequence information is of great importance. In this study, fat body-specific transcriptome analysis revealed a surprising number of transcripts implicated in the immune response. A total of 71 unigenes encoding various immune-related enzymes were identified, which corresponded to 25 serine proteinase inhibitors or serpins, 17 immuno- and C-type lectins, 11 transferrins, six peptidoglycan recognition proteins (PGRPs), three lysozymes, three defensins, three gram-negative bacteria-binding proteins (GNBP), two β-1,3-glucan recognition proteins (β-GRP), and one defense protein Hdd11-like homolog.

**Figure 3:**
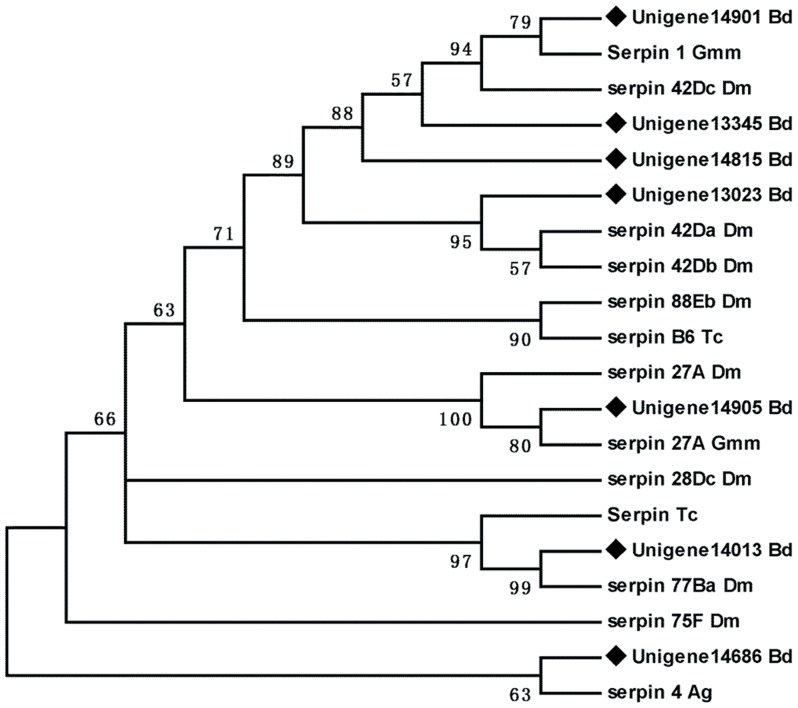
Phylogenetic analysis of serpins from *Bactrocera dorsalis* (Bd), *Drosophila melanogaster* (Dm), *Anopheles gambiae* (Ag), *Tribolium castaneum* (Tc) and *Glossina morsitans morsitans* (Gmm). The tree was created using the neighbor-joining method. Bootstrap values represent the percentage of 1000 replicates. Reference sequences were obtained from the NCBI database.

The most striking group is the 25 unigenes corresponding to putative serine proteinase inhibitors or serpins. Among these sequences, 10 matched known *B. dorsalis* serpins, and other 15 unigenes were novel. Serpins are irreversible inhibitors of serine proteases that regulate various physiological and pathological reactions in humans and insects, especially in innate immune responses [17]. To date, multiple serpin genes have been identified in insect genomes [18]. Previous studies have shown that the serpin genes were highly enriched in the fat body, reflecting this organ's involvement in the immune response of insects [19]. Here, we analyzed the carboxyl-terminal inhibitor domain of seven complete sequences of serpins in the fat body of *B. dorsalis*. Phylogenetic analysis showed that the seven were classified into different clades (Figure 3). In serpin proteins, there is a highly conserved tertiary structure with a reactive center loop region near the C-terminus, which acts as the bait for the target protease [20]. Functional analyses of serpins in *Manduca sexta* found that there were also seven groups of serpin proteins: most of them are inhibitory and regulate proteases that have a role in cascades leading to activation of prophenoloxidase (proPO) and the cytokine spätzle [19]. In addition, *Mamestra configurata* serpin-1 homologs, *serpins-1b* and *serpins-1c*, are possibly involved in developmental processes related to the molt [21]. The multiple serpin family genes may have diverse functions. Therefore, further investigations are needed to determine the roles of these serpins in *B. dorsalis*.

**Figure 4:**
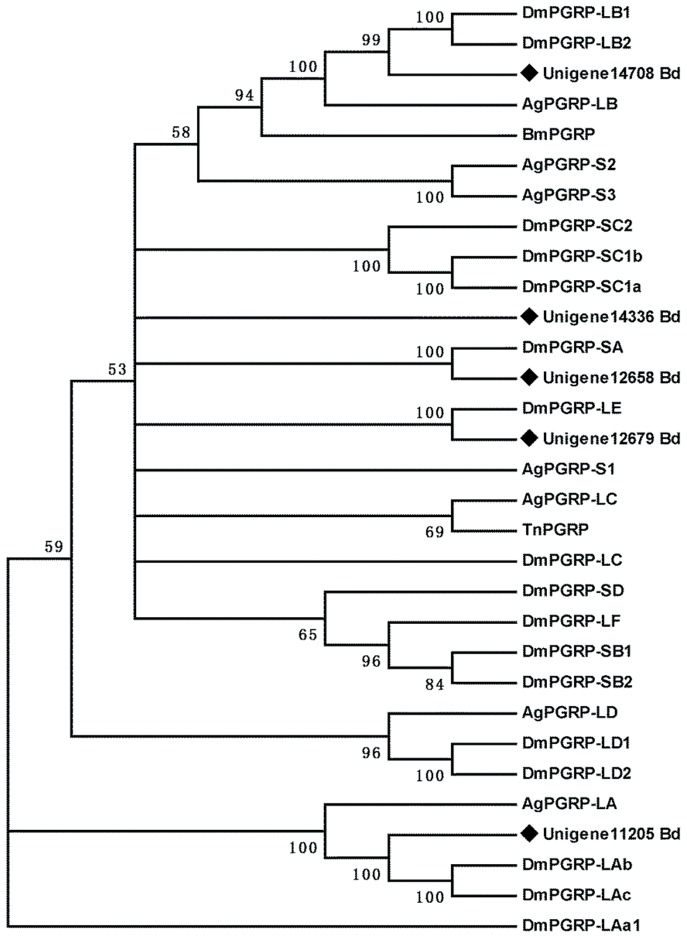
Phylogenetic analysis of peptidoglycan recognition proteins from *Bactrocera dorsalis* (Bd), *Drosophila melanogaster* (Dm), *Anopheles gambiae* (Ag), *Bombyx mori* (Bm) and *Trichoplusia ni* (Tn). The tree was created using the neighbor-joining method. Bootstrap values represent the percentage of 1000 replicates. Reference sequences were obtained from the NCBI database.

In the insect immune system, PGRPs are important pattern recognition molecules that recognize bacteria and their unique cell wall component, and are conserved from insects to mammals [22]. Based on structural analyses, insect PGRPs can be classified into two different types: short (PGRP-S) and long (PGRP-L) [23]. PGRP-Ss are small extracellular proteins, while PGRP-Ls are long intracellular or membrane-spanning proteins. Insect PGRPs have various functions that are unique to insects, including activation of the proPO cascade, Toll receptor and *Imd* pathways, as well as induction of phagocytosis [24]. In this study, six complete sequences encoding PGRPs with an average length of 1001 bp were identified from the fat body of *B. dorsalis*. Phylogenetic analysis together with genes from *D. melanogaster* and *Anopheles gambiae* showed that three could be assigned to the PGRP-L group and the other two were assigned to the PGRP-S group (Figure 4). The functional PGRP groups form discrete branches on phylogenetic tree of insect PGRPs: thus, the amino acid sequences of PGRPs correlate with the functions of PGRPs, permitting us to form an initially prediction of the functions of the PGRPs in *B. dorsalis*. In addition to PGRPs, GNBP and β-GRP are also important pattern recognition molecules. In *Nilaparvata lugens*, significantly higher expression levels of these two types of genes were observed in fat bodies compared with other tissues, which is consistent with their role in immunity [25]. Here we identified three GNBP genes and two beta-GRP genes in the fat body of *B. dorsalis* for the first time.

**Figure 5:**
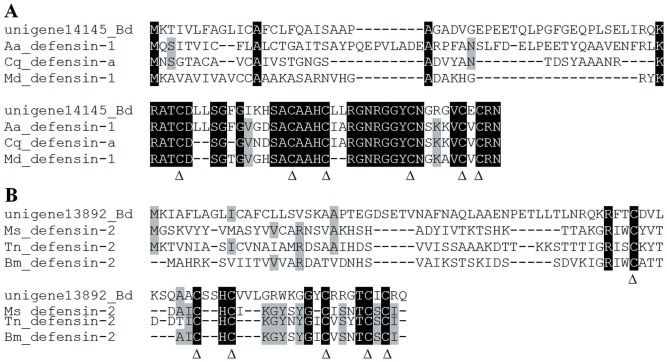
Amino acid alignment of two new defensin-like proteins in *Bactrocera dorsalis*. The ClustalW multiple alignment program aligned the amino acid sequences. Identical residues are shown in white with black background. The defensin family motif and six highly conserved residues are indicated with a triangle. *Aedes aegypti* (Aa), *Bactrocera dorsalis* (Bd), *Bombyx mori* (Bm), *Culex quinquefasciatus* (Cq), *Musca domestica* (Md), *Manduca sexta* (Ms), *Trichoplusia ni* (Tn).

Defensins, a class of small and diverse cysteine-rich proteins, have broad-spectrum antimicrobial activity against bacteria, fungi and enveloped viruses [26]. Here, we newly identified three defensin-like protein genes in the fat body of *B. dorsalis*. We compared the predicted amino acid sequences of these three new defensin-like proteins, termed defensin-like-1, -2 and -3, with other insect defensins. Two of them represented full length of open reading frames (ORFs), whereas the defensin-like-3 lacked a coding sequence at the 5′ end. Furthermore, multiple protein alignments showed that the two complete defensin-like proteins have six highly conserved cysteine residues (Figure 5). Such defensin-like proteins were also identified in the midgut-specific transcriptomes of *M. sexta* and *Plutella xylostella* [27], [28].

**Figure 6:**
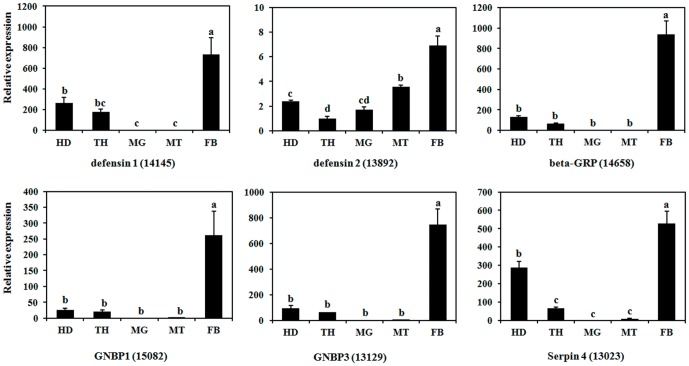
Tissue specificity of immunity-related genes expression in *Bactrocera dorsalis*. Expression levels in head (HD), thorax (TH), midgut (MG), Malpighian tubules (MT) and fat body (FB) were detected by qPCR. The relative expression was calculated based on the value of the lowest expression, which was ascribed an arbitrary value of 1. Different letters above the bars indicate significant differences based on Duncan's new multiple range tests (DMRT, *P*<0.05).

To determine whether these genes are related to the immune response, we randomly selected six genes to characterize their expression patterns in different tissues. Interestingly, all the selected genes demonstrated significantly higher expression levels in the fat body compared with other tissues (Figure 6). The six genes showed fat body-specific expression, which implied that the fat body is the primary site of immunity-related genes expression. These results will provide comprehensive information to enable the determination of the molecular mechanism underlying the immune response in *B. dorsalis*.

### Detoxification-related genes

In the last decade, *B. dorsalis* has become a serious problem in fruit production, and has developed resistance to various insecticides [2]. The insect fat body, classically viewed as a tissue primarily involved in xenobiotic metabolism, harbors several detoxification enzymes [9], [11]. In this study, we obtained abundant transcripts in the fat body of *B. dorsalis*, especially the unigenes encoding cytochrome P450, glutathione S-transferases and ATP-binding cassette transporters, which were mainly involved in detoxification of xenobiotic [29], [30].

### Cytochrome P450 (P450s)

**Figure 7:**
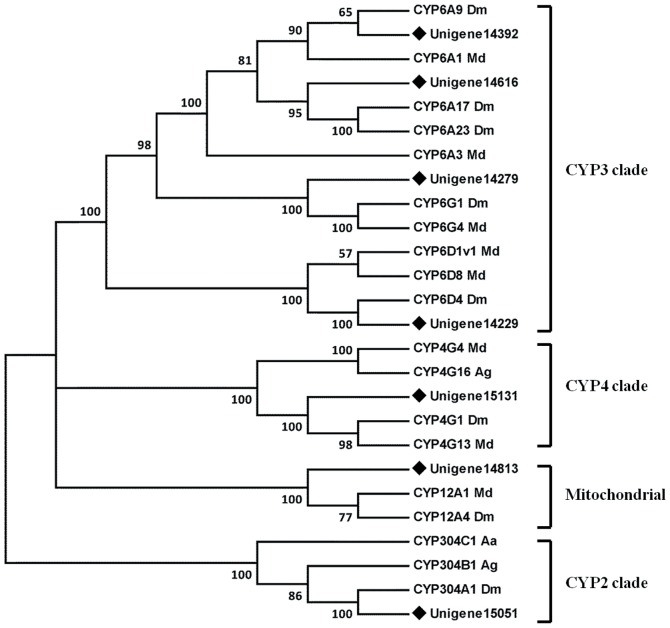
Phylogenetic analysis of cytochrome P450s from *Bactrocera dorsalis* (Bd), *Drosophila melanogaster* (Dm), *Musca domestica* (Md), *Anopheles gambiae* (Ag) and *Aedes aegypti* (Aa). The tree was created using the neighbor-joining method. Bootstrap values represent the percentage of 1000 replicates. Reference sequences were obtained from the NCBI database.

P450s compose a superfamily that is involved mainly in the metabolism of a variety of endogenous and exogenous compounds [31]. In this study, 37 distinct sequences with an average length of 1089 bp encoding P450s were identified. Based on the closest BLAST hit in the NCBI nr database, these unigenes were divided into four clades and 14 families, including four families of CYP18, CYP 304, CYP305 and CYP307 in the CYP2 clade; three families of CYP6, CYP9, CYP309 and CYP317 in the CYP3 clade, two families of CYP4 and CYP311 in the CYP4 clade; and four families of CYP12, CYP301, CYP302 and CYP314 in the mitochondrial CYP clade (Table 2). Interestingly, 17 of these unigenes predicted novel P450 genes in the fat body of *B. dorsalis*. Among these, seven unigenes appeared to be full length, and were further subjected to phylogenetic analysis with other known insect P450s. The results showed that four unigenes belonged to the CYP3 clade, and one each to the CYP4, CYP2 and mitochondrial CYP clades (Figure 7).

**Table 2:** Different CYPs P450 clades, families and GSTs classes in the fat body transcriptome of *Bactrocera dorsalis*.

Enzymes	Occurrence	Family members with corresponding number
**CYPs P450**		
***CYP2 clade*** ** (4 family)**		
CYP18	2	*CYP18A1* (2)
CYP304	3	*CYP304A1* (3)
CYP305	1	*CYP305A1* (1)
CYP307	3	*CYP307A1* (3)
***CYP3 clade*** ** (3 family)**		
CYP6	30	*CYP6A2* (1), *CYP6A9* (6), *CYP6A13* (4), *CYP6A14* (4), *CYP6A21* (1), *CYP6A22* (1), *CYP6D4* (1), *CYP6D*5 (1), *CYP6G1* (5), *CYP6U1* (3), *CYP6V1* (3)
CYP9	6	*CYP9B2* (3), *CYP9F2* (1), *CYP9H1* (2)
CYP309	4	*CYP309A1* (4)
CYP317	3	*CYP317A1* (3)
***CYP4 clade*** ** (2 family)**		
CYP4	20	*CYP4AC1* (3), *CYP4AE1* (1), *CYP4D1* (2), *CYP4D2* (1), *CYP4E1* (2), *CYP4E2* (1), *CYP4G1* (5), *CYP4P1* (3), *CYP4S3* (2)
CYP311	2	*CYP311A1* (2)
***Mitochondrial CYP clade*** ** (4 family)**		
CYP12	15	*CYP12A4* (7), *CYP12B1* (2), *CYP12B2* (4), *CYP12C1* (2)
CYP301	1	*CYP301A1* (1)
CYP302	4	*CYP302A1* (4)
CYP314	1	*CYP314A1* (1)
**GSTs classes**		
Delta	9	Delta (3), Delta1 (3), Delta2 (1), Delta5 (1), Delta6 (1)
Epsilon	5	Epsilon2 (1), Epsilon4 (1), Epsilon5 (2), Epsilon9 (1)
Omega	3	Omega1 (3)
Sigma	1	Sigma1 (1)
Theta	3	Theta1 (1), Theta3 (1), Theta5 (1)
Microsomal	3	Microsomal (2), Microsomal-like (1)
Zeta	3	Zeta1 (1), Zeta2 (2)

In the present study, more than half of the P450s belonged to the CYP3 and CYP4 clades, which is in accordance with data from other insects [25], [28]. CYP3 and CYP4 in other insects are also the most abundant P450 clades, and their function is related to xenobiotic metabolism [31]. For example, overexpression of *CYP 6G1* in *D. melanogaster* resulted in the increased metabolic detoxification of insecticides [32]. In addition, our previous study of P450s in *B. dorsalis* identified nine genes that are mainly expressed in the fat body, and were upregulated in response to insecticide exposure [33]. This suggested that P450s detoxification probably takes place mainly in the fat body of *B. dorsalis*. Therefore, we believe that the new P450 genes identified here may contribute to insecticide resistance. Further investigations are required to determine the roles of these novel P450 genes in *B. dorsalis*.

### Glutathione S-transferases (GSTs)

**Figure 8:**
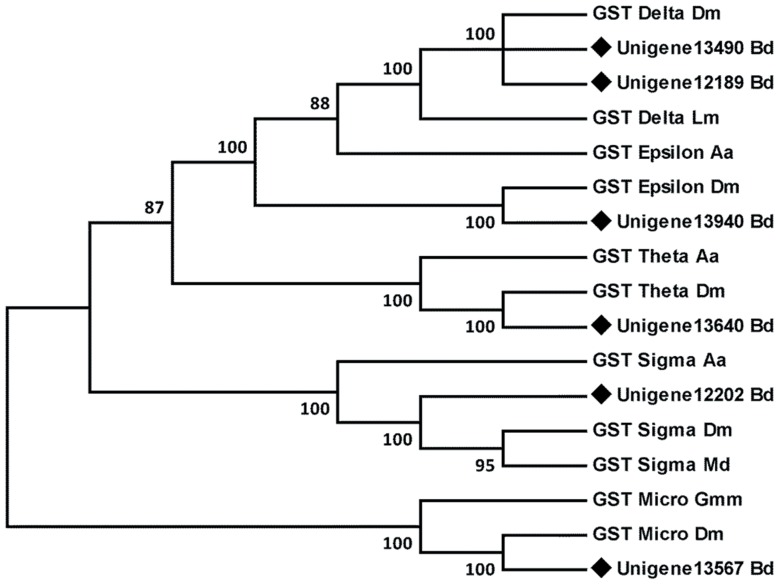
Phylogenetic analysis of the glutathione S-transferases from *Bactrocera dorsalis* (Bd), *Drosophila melanogaster* (Dm), *Musca domestica* (Md), *Aedes aegypti* (Aa), *Locusta migratoria* (Lm) and *Glossina morsitans morsitans* (Gmm). The tree was created using the neighbor-joining method. Bootstrap values represent the percentage of 1000 replicates. Reference sequences were obtained from the NCBI database.

GSTs are a group of multifunctional enzymes that play important roles in the detoxification of hydrophobic toxic compounds as well as mediating the oxidative stress response [34]. After removing redundant sequences, 18 different sequences with an average length of 762 bp encoding specific GSTs were identified in the fat body of *B. dorsalis* (Table 2). These GSTs were assigned to the delta, epsilon, sigma, theta, zeta and microsomal classes. Among them, 10 correspond to known *B. dorsalis* GSTs, and the other eight sequences were newly reported. Specifically, six of the new GSTs appeared to be complete, and these sequences were further subjected to phylogenetic analysis with other known insect GSTs. The results showed that two unigenes belonged to the delta class, and one unigene was assigned to each of the epsilon, theta, sigma and microsomal GST classes (Figure 8).

Half of GSTs identified in the fat body of *B. dorsalis* belong to the delta and epsilon classes, members of which are insect-specific and are implicated mainly in insecticide resistance and detoxification [35]. Earlier studies reported that these two classes of GSTs were responsible for organophosphate, DDT, and pyrethroid resistance [34], [36]. Interestingly, we identified complete sequences of two delta and one epsilon; these genes appear to be important in the detoxification of various insecticides. Notably, compared with our previous transcriptomes of *B. dorsalis*, we found a new sigma gene in the fat body. Evidence suggests that insect sigma GSTs are not only involved in detoxification of exogenous compounds and oxidative stress resistance, but also probably play a role in muscle structure [37], [38]. Hence, the sigma GST in *B. dorsalis* is likely to have multiple functions. It is generally presumed that GST detoxification occurs mainly in the insect fat body [34]. We identified several GSTs in the fat body of *B. dorsalis*; however, the detailed functions of these genes remain to be elucidated.

### ATP-binding cassette (ABC) transporters

**Figure 9:**
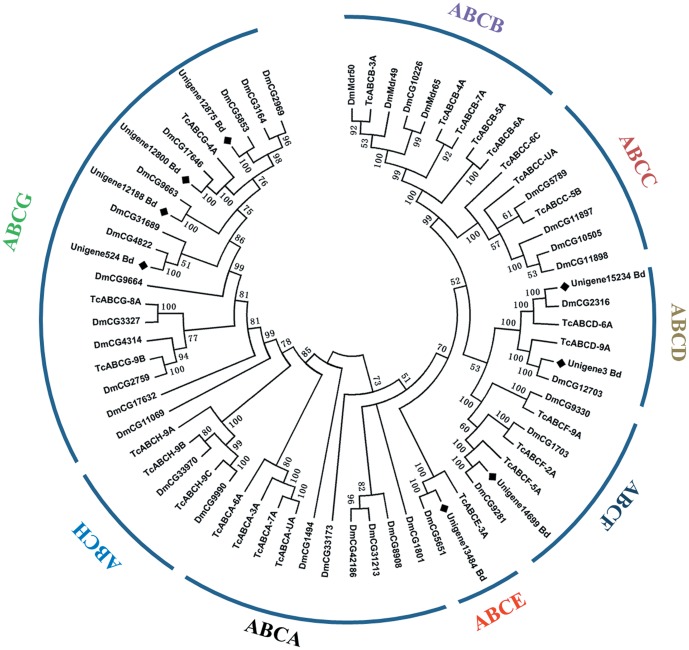
Phylogenetic analysis of ABC transporters from *Bactrocera dorsalis* (Bd), *Drosophila melanogaster* (Dm) and *Tribolium castaneum* (Tc). The tree was created using the neighbor-joining method. Bootstrap values represent the percentage of 1000 replicates. Reference sequences were obtained from the NCBI database.

In insects, ABC transporters are not only responsible for transporting molecules, but also are involved in the biochemical defense against toxicants [39]. From the fat body transcriptome, 29 different sequences with an average length of 1467 bp encoding ABC transporters were identified; and no allelic variants were found. These sequences were classified into seven subfamilies (four belong to A subfamily, four belong to B subfamily, one belongs to C subfamily, two belong to D subfamily, one belongs to E subfamily, three belong to F subfamily, and four belong to G subfamily) (Table S2). Among these, eight sequences had a complete ORF and were subjected to phylogenetic analysis with *D. melanogaster* ABC transporters. We found that two belong to the D subfamily, one each to the E and F subfamilies, and four to the G subfamily (Figure 9).

Analyses of fully sequenced insect genomes have identified 68 ABC transporters in *Tribolium castaneum*, 56 in *D. melanogaster*, 52 in *A*. *gambiae*, 51 in *Bombyx mori*, and 43 in *Apis mellifera* (Table S2). Additionally, 18 ABC transporters were identified in the *B. oleae* transcriptome [40]. The numbers of ABC transporters were compared among seven different insect species; the 68 genes in *T. castaneum* form the largest ABC transporter data set, but the number of ABC transporters identified in this study is greater than that in *B. oleae*. The number of ABC transporters in the fat body of B. dorsalis is within the range of ABC transporters in other insect species (18-69) (Table S2), additional ABC transporters may await discovery because of their absence from the current transcriptomic database. In *D. melanogaster*, the ABC transporter was regulated by heavy metals via the metal-responsive transcription factor 1 and are associated with biochemical detoxification of zinc and copper [41]. Recently, three Lepidopteran ABC transporter subfamilies, including B, C, and G, were reported to confer resistance to xenobiotics, such as insecticides [30]. Therefore, over half of the ABC transporters identified in this study are believed to be involved in detoxification, which represent promising avenues for further investigation.

**Figure 10:**
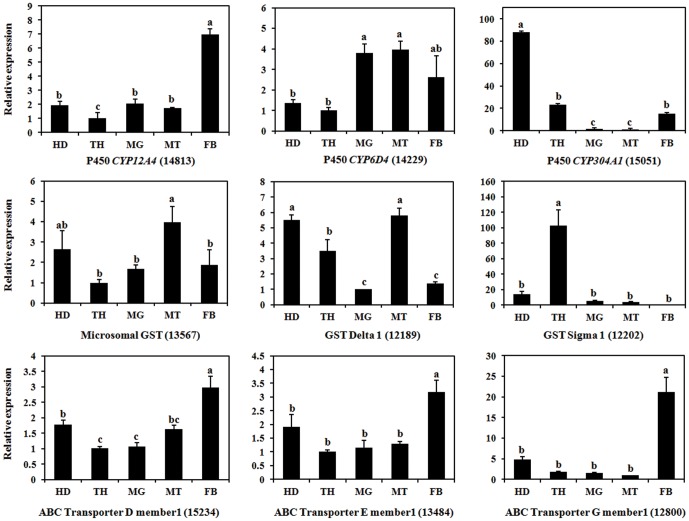
Tissue specificity of detoxification-related genes expression in *Bactrocera dorsalis*. The qPCR analysis was performed as described in Figure 6.

We randomly selected nine detoxification-related genes to determine their expression specificity. Four genes showed higher expression levels in the fat body than in other tissues, including three ABC transporters, D member 1 (unigene 15234), E member 1 (unigene 13484), G member 1 (unigene 12800), and *CYP12A4* (unigene 14813), and displayed tissue-specific expression patterns (Figure 10). In contrast, the remaining three GSTs and two P450s showed low expression levels in the fat body. The exclusive expression of several genes in the fat body indicates that these detoxification-related genes probably play vital roles in the metabolism of xenobiotics, and thus permit substantial molecular studies of the mechanism of detoxification in *B. dorsalis*.

### Genes putatively involved in energy metabolism

The insect fat body, equivalent to vertebrate adipose tissue and liver, is a major organ involved in energy metabolism [10], [42]. During insect growth and development, insects store the energy reserves in the fat body in response to energy demands. It is generally accepted that energy is crucial for the activity and movement of organisms, and disruption of energy homeostasis affects natural development and vital movement, leading to eventual death of organisms. Therefore, identification of genes related to energy metabolism is significant and would help develop new methods to control pest insects via interference with the energy system. In this study, many unigenes were identified that share high similarity with genes related to energy metabolism in other insects, including fatty acid desaturase, lipase, alpha-amylase, trehalose-6-phosphate synthase, ATP synthase, glycerol-3-phosphate dehydrogenase, aldose 1-epimerase, beta-glucosidase, ribosomal protein S7 and aminopeptidase (Table 3).

**Table 3:** Details of sequences involved in energy metabolism.

	Unigene ID	Length	Homology genes in Nr/Gene ID	Homology species	E-value
Fatty acid desaturase	Unigene14007	2136	fatty acid desaturase/AAB17283	*Drosophila melanogaster*	0
	Unigene14769	446	fatty acid desaturase 2/BAB21537	*Drosophila melanogaster*	3.00E^−72^
Lipase	Unigene4378	1296	monoacylglycerol lipase ABHD12-like isoform 1/XP_001604091	*Nasonia vitripennis*	1.00E^−60^
	Unigene13291	784	lipase 4, isoform A/NP_609418	*Drosophila melanogaster*	2.00E^−82^
	Unigene14677	1540	brummer lipase/ATGL-like protein, partial/	*Glossina morsitans morsitans*	0
	Unigene13974	1382	lipase 3-like/XP_003695770	*Apis florea*	2.00E^−62^
Alpha-amylase	Unigene12619	1059	alpha-amylase 3/EFN65423	*Camponotus floridanus*	2.00E^−50^
	Unigene14089	1814	alpha-amylase/XP_001649784	*Aedes aegypti*	1E^-174^
Trehalose-6-phosphate synthase	Unigene15155	2402	trehalose-6-phosphate synthase/NP_608827	*Drosophila melanogaster*	0
ATP synthase	Unigene9638	671	V-ATPase G subunit/AEV43310	*Bactrocera dorsalis*	9.00E^−38^
	Unigene13304	1909	vacuolar ATP synthase subunit S1/ABF18127	*Aedes aegypti*	4.00E^−33^
	Unigene429	2252	vacuolar ATP synthase subunit E/NP_001040451	*Bombyx mori*	1.00E^−92^
	Unigene14983	2480	ATP synthase alpha subunit vacuolar/XP_001849275	*Culex quinquefasciatus*	0
	Unigene12971	2456	ATP synthase/EHJ65007	*Danaus plexippus*	0
	Unigene9659	765	ATP synthase delta chain, mitochondrial/EAT46274	*Aedes aegypti*	3.00E^−48^
	Unigene14297	600	ATP synthase F0 subunit 6/YP_006883645	*Bactrocera tryoni*	2.00E^−55^
	Unigene455	784	H+ transporting ATP synthase O subunit isoform 1/NP_001040526	*Bombyx mori*	1.00E^−62^
Glyceraldehyde-3-phosphate dehydrogenase	Unigene11633	981	glyceraldehyde-3-phosphate dehydrogenase/ADO24527	*Bactrocera dorsalis*	6.00E^−157^
Aldose 1-epimerase	Unigene3995	327	aldose 1-epimerase/XP_001843677	*Culex quinquefasciatus*	2.00E^−40^
Beta-glucosidase	Unigene14223	1153	beta-glucosidase/XP_001659855	*Coptotermes formosanus*	2.00E^−84^
Ribosomal protein S7	Unigene12342	555	ribosomal protein S7, isoform A/NP_651782	*Drosophila melanogaster*	1.00E^−87^
	Unigene12887	772	mitochondrial ribosomal protein S7, isoform A/NP_523537	*Drosophila melanogaster*	6.00E^−71^
Aminopeptidase N	Unigene6316	630	Aminopeptidase N/EFN68544	*Camponotus floridanus*	2.00E^−41^
Aminopeptidase P	Unigene15293	1954	aminopeptidase P, isoform A/NP_477409	*Drosophila melanogaster*	0

The fatty acid desaturase gene, which encodes a predicted protein mainly expressed in the fat body, plays a pivotal role in mitochondrial energy metabolism as well as controlling body size [43]. In *D. melanogaster*, mutation in this gene can induce a sustained change in energy homeostasis [44]. Here we identified one *B. dorsalis* fatty acid desaturase (unigene14007, 2,136 bp), which is similar to the same functional gene in *D. melanogaster* (AAB17283).

Lipids represent the major component of the fat body and are the main source of metabolic fuel [45]. Lipid metabolism is required to meet the energy demand during non-feeding periods. Brummer lipase, or insect adipose triglyceride lipase (ATGL), belongs to the calcium-independent phospholipase A_2_ (iPAL_2_) family, and plays an important role in energy metabolism [46]. In *D. melanogaster*, loss of *ATGL* caused accumulation of triglyceride, leading to fat flies, whereas its overexpression produced lean flies [47]. In the fat body of *B. dorsalis*, we found unigene 14677 (1,540 bp) that was a full-length sequence encoding an *ATGL*, which showed similarity with a specific gene of *G. morsitans morsitans*, whose function was not annotated.

Alpha-amylase is a common enzyme that catalyzes hydrolysis of α-(1,4) glycosidic linkages in starch and related compounds to serve as an energy source [48]. This enzyme can encounter varying proportions of starch constituents in the diet to provide the energy required for all cellular functions [49]. In the present study we identified two sequences encoding alpha-amylase in the fat body of *B. dorsalis*, for the first time.

In insects, the blood sugar is trehalose instead of glucose; therefore, the synthesis and utilization of trehalose is unique to insect energy metabolism compared with other animals [50]. Trehalose-6-phosphate synthase (TPS) is the crucial enzyme for the biosynthesis of trehalose, and catalyzes the formation of trehalose-6-phosphate from uridine diphosphate glucose and glucose-6-phosphate [51]. Apart from the synthesizing trehalose in insects, TPS was also reported to be an important enzyme for insect development. For example, mutations of the *TPS* gene in *D. malanogaster* lead to early larval lethality and affected normal insect development [52]. Furthermore, the development of *N. lugens* and *Spodotera exigua* larvae treated with dsRNA of *TPS* was disturbed, resulting in lethality, suggesting that a potential way to control the pests is to disrupt *TPS* expression using RNAi [53], [54]. In the fat body of *B. dorsalis*, we found a 2,402 bp sequence (unigene 15155) encoding TPS, which showed similarity the same functional gene of *D. melanogaster* (NP_608827). Taken together, a better understanding of the proteins related to energy metabolism will help to develop new methods to control this insect pest.

**Figure 11:**
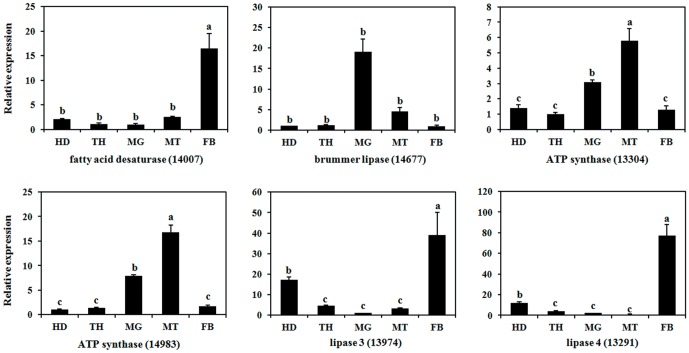
Tissue specificity of energy metabolism-related genes expression in *Bactrocera dorsalis*. The qPCR analysis was performed as described in Figure 6.

In this study, we randomly selected six genes similar to fatty acid desaturase, lipase, and ATP synthase to analyze their expression differences among different tissues, and to detect whether these genes are involved in energy metabolism in *B. dorsalis*. The qPCR analysis showed significantly higher expression levels of two lipase genes, lipase 3 (unigene 13974) and lipase 4 (unigene 13291), and fatty acid desaturase (unigene 14007) in the fat body; much lower levels were observed in the head (Figure 11). Additionally, two ATP synthase genes were both mainly expressed in Malpighian tubules, while brummer lipase (unigene 14677) was mainly expressed in the midgut. Our results may reflect the functional diversification of these genes, thus providing a valuable resource for further studies on energy metabolism in *B. dorsalis*.

## Conclusions

In this study, we used Illumina sequencing technology to investigate the fat body transcriptome of *B. dorsalis*. We assembled the raw reads into 27,787 unigenes with an average length of 591 bp. Many genes that are potentially relevant to immunity, detoxification and energy metabolism were identified within the annotated unigenes. Specific information for these unigenes identified in this study is shown in Table S4. Several genes showed fat body-specific expression, implying that these genes might be potential targets for *B. dorsalis* management. Meanwhile, the fat body-specific transcriptome analysis generated a large number of genes newly identified in *B. dorsalis*, which will facilitate further functional study. These results provided basic information to enhance our understanding of physiological and immunological mechanism that occur in the *B. dorsalis* fat body, and laid the foundation for future functional genomics studies.

## Materials and Methods

### Ethics Statement

No specific permits were required for the insects collected in this study. No specific permissions were required for these locations/activities which the insect specimens were collected. We confirm that these locations are not privately-owned or protected in any way and the species collections did not involve endangered or protected species.

### Insect samples

The stock colony of *B. dorsalis* was originally collected from Fujian province, China. The insects were reared under laboratory conditions at 27±1°C, 70±5% relative humidity, and a 14:10 h light: dark photoperiod, using an artificial diet, as described previously [55].

### Collection of fat bodies

To obtain complete gene expression information, fat bodies of *B. dorsalis* were collected from different developmental stages, including the third-instar larvae, pupae and adults. The insects were anesthetized on ice for 30 min and dissected under a stereomicroscope (Olympus SZX12, Tokyo, Japan). The samples were isolated on ice, placed in a 2.0 mL DEPC-treated centrifuge tube containing RNA store reagent (TIANGEN, Beijing, China), placed immediately in liquid nitrogen and frozen at −80°C.

### RNA isolation for transcriptome analysis

An RNeasy plus Micro Kit (QIAGEN, Hilden, Germany) extracted the total RNA from each sample, according to manufacturer's instructions, and gDNA Eliminator spin columns removed the genomic DNA. Measuring the absorbance at 260 nm using a NanoVue UV-Vis spectrophotometer (GE Healthcare Bio-Science, Sweden) quantified the RNA. The absorbance ratio of OD_260/280_ and OD_260/230_ was used to assess the purity of the RNA and 1% agarose gel electrophoresis confirmed the integrity of the RNA. A mixture of RNA from all these developmental stages at an equal ratio was used to construct the whole-set transcriptome.

### Illumina sequencing analysis

The cDNA library of *B. dorsalis* fat body was constructed using an mRNA-Seq assay for paired-end transcriptome sequencing. The Beijing Genomics Institute (BGI, Shenzhen, China) performed the sequencing. Briefly, poly (A) mRNA was purified from 20 μg of total RNA using Oligo (dT) magnetic beads and then fragmented into short sequences in the presence of fragmentation buffer at 94°C for 5 min. These short fragments were used as templates for first-strand cDNA synthesis using random hexamer-primers. Subsequently, second-strand cDNA synthesis was performed using buffer, dNTPs, RNaseH, and DNA polymerase Ι. A QiaQuick PCR extraction kit (Qiagen) purified the short fragments, which were then washed with EB buffer for end reparation and single nucleotide adenine addition. After the end-repair and ligation of adaptors, PCR amplified the cDNA products to create a fat body specific sequencing library. The cDNA library was sequenced on an Illumina HiSeq 2000 instrument for 4 gigabase in-depth in a single run.

### *De novo* assembly

Transcriptome *de novo* assembly was carried out using the short reads assembling program Trinity [56]. Firstly, reads with a certain length of overlap were combined to form longer fragments without Ns, forming contigs. Then, using paired-end reads, contigs from the same transcript, as well as the distances between these contigs were detected. Finally, Trinity connected these contigs to produce consensus sequences that could not be extended at either end, and were defined as unigenes. After clustering, the obtained unigenes were divided into two classes: clusters and singletons. The distinct sequences were compared to protein databases including nr, Swiss-Prot, KEGG and COG using the BLASTx algorithm with a significant cut-off *E*-value of <10^−5^. If the alignment results of different databases conflicted with each other, we followed the priority order of Nr, Swiss-Prot, KEGG, and COG when determining the unigene sequence direction. Blast2GO software with default parameters performed annotation with gene ontology terms (GO, http://www.geneontology.org). Blastall software against Cluster of Orthologous Groups database and Kyoto Encyclopedia of Genes and Genomes database performed the COG and KEGG pathway annotations, respectively.

### Identification and analysis of interesting genes

Interesting sequences were identified from the BLAST results generated against the nr database with a cut-off *E*-value of <10^−5^. ORF finder (http://www.ncbi.nlm.nih.gov/gorf/gorf.html) determined the complete coding region and protein BLAST results were generated. MEGA 5.04 [57] was used to analyze the phylogenetic relationships between Serpins, PGRPs, P450, GSTs, and ABC transporter genes and those from other insects to infer their classification. The neighbor-joining method (with pairwise deletion) was used to create the phylogenetic trees. A bootstrap test was conducted (1000 replications) to calculate the percentage of replicate trees in which sequences clustered together. ClustalW software generated the multiple alignments of the various protein sequences [58].

### Quantitative real-time PCR (qPCR) analysis

Different tissues, including the head, thorax, midgut, Malpighian tubules and fat body, were dissected from day-7 adults to investigate tissue-specific gene expression patterns. The TRIzol reagent (Invitrogen, Carlsbad, CA, USA) was used to extract total RNA from each tissue sample, which was then treated with DNase (TaKaRa, Dalian, China) for DNA digestion. RNAs (1 μg) were reverse transcribed into first-strand cDNAs using PrimeScript RT reagent Kit (TaKaRa, Dalian, China). The qPCR reactions were performed in a 20 μL total reaction volume including 10 μL GoTaq qPCR Master Mix (Promega, Madison, WI, USA), 1 μL of template cDNA, 7 μL ddH2O, and 1 μL of each primer (0.2 mM). The reaction was carried out on an ABI 7500 Real-Time PCR System (Applied Biosystems, Foster City, CA, USA) under the following thermal program: 1 cycle of 95°C for 2 min, followed by 40 cycles of 95°C for 15 s and 60°C for 30 s. At the end of each qPCR experiment, a melting curve was generated to rule out the possibility of primer-dimer formation. The sequences of the specific primer sets are listed in Table S3. The *α-Tubulin* (GU269902) gene from *B. dorsalis* was used as an internal control. The relative expression levels were calculated using 2^−ΔΔCt^ method [59]. Results were expressed as the mean ± SE of three biological replications. Data were analyzed statistically using one-way analysis of variance (ANOVA), and the means were separated by Duncan's multiple range tests for significance (*P*<0.05) using SPSS 16.0 for windows (SPSS Inc., Chicago, IL, USA).

## Supporting Information

Figure S1 **Size distribution of unigenes in the fat body of** ***Bactrocera dorsalis*** **.** The sizes of 27,787 unigenes that had a BLAST annotation in the NCBI database were calculated.
(TIF)

Figure S2 **Homology analysis of unigenes in the fat body of** ***Bactrocera dorsalis*** **.** All distinct gene sequences that had BLAST annotations against the nr database with a cut-off *E*-value of 1.0E^−5^ were analyzed. The sequences were summarized based on the (A) *E*-value distribution, (B) similarity distribution, and (C) species distribution, respectively.
(TIF)

Table S1 **KEGG annotation of unigenes.**
(XLSX)

Table S2 **Comparatively analysis of ABC transporters in seven insect species.**
(XLSX)

Table S3 **Primers used in real-time qPCR for tissue specific expression.**
(XLSX)

Table S4 **Sequence information of all unigenes identified in this study.**
(XLSX)
