# Supplementary figures and images for: De novo Cloning and Annotation of Genes Associated with Immunity, Detoxification and Energy Metabolism from the Fat Body of the Oriental Fruit Fly, Bactrocera dorsalis
Source: PLoS One. 2014 Apr 7;9(4):e94470. doi: 10.1371/journal.pone.0094470 (PMC3978049; doi:10.1371/journal.pone.0094470)

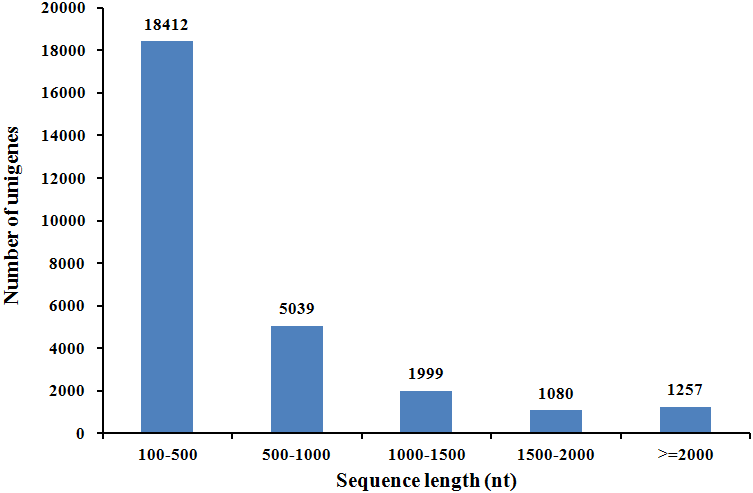

Supplement: Figure S1 — Size distribution of unigenes in the fat body of Bactrocera dorsalis . The sizes of 27,787 unigenes that had a BLAST annotation in the NCBI database were calculated. (TIF) [file pone.0094470.s001.tif]

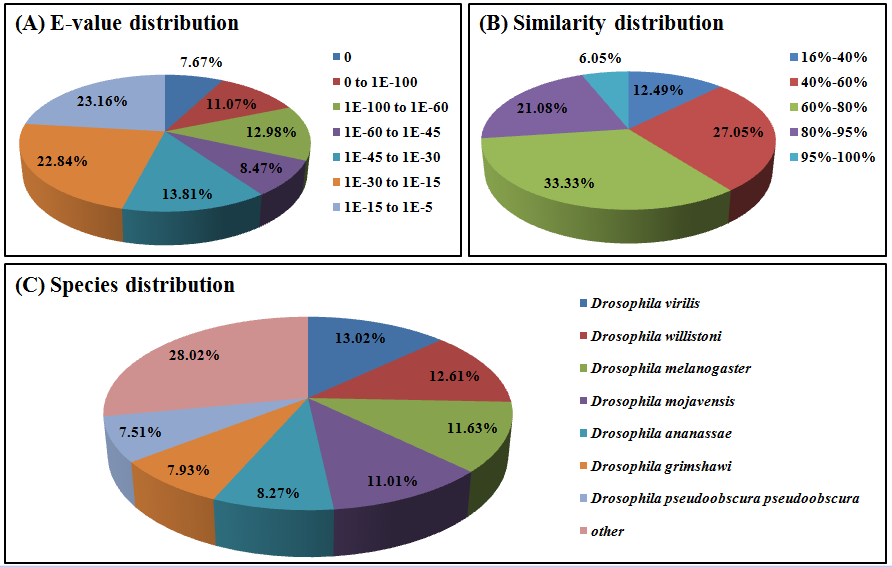

Supplement: Figure S2 — Homology analysis of unigenes in the fat body of Bactrocera dorsalis . All distinct gene sequences that had BLAST annotations against the nr database with a cut-off E-value of 1.0E−5 were analyzed. The sequences were summarized based on the (A) E-value distribution, (B) similarity distribution, and (C) species distribution, respectively. (TIF) [file pone.0094470.s002.tif]
